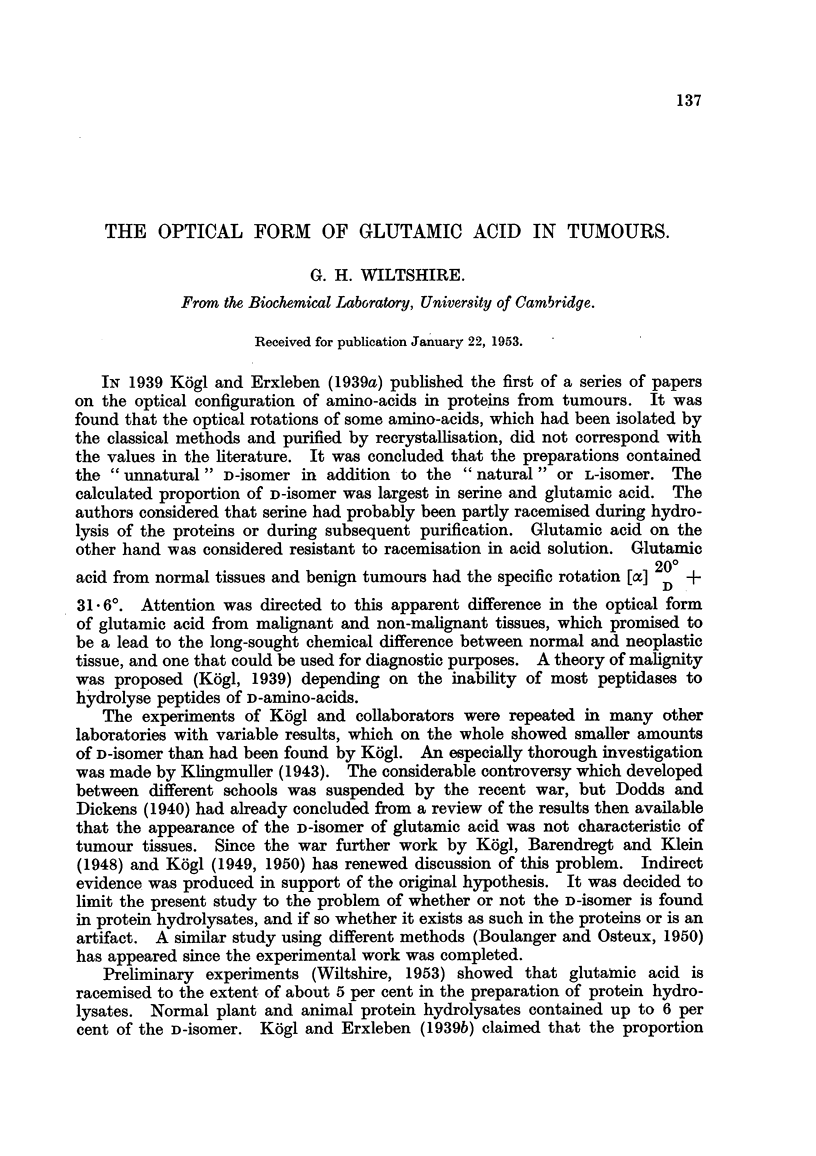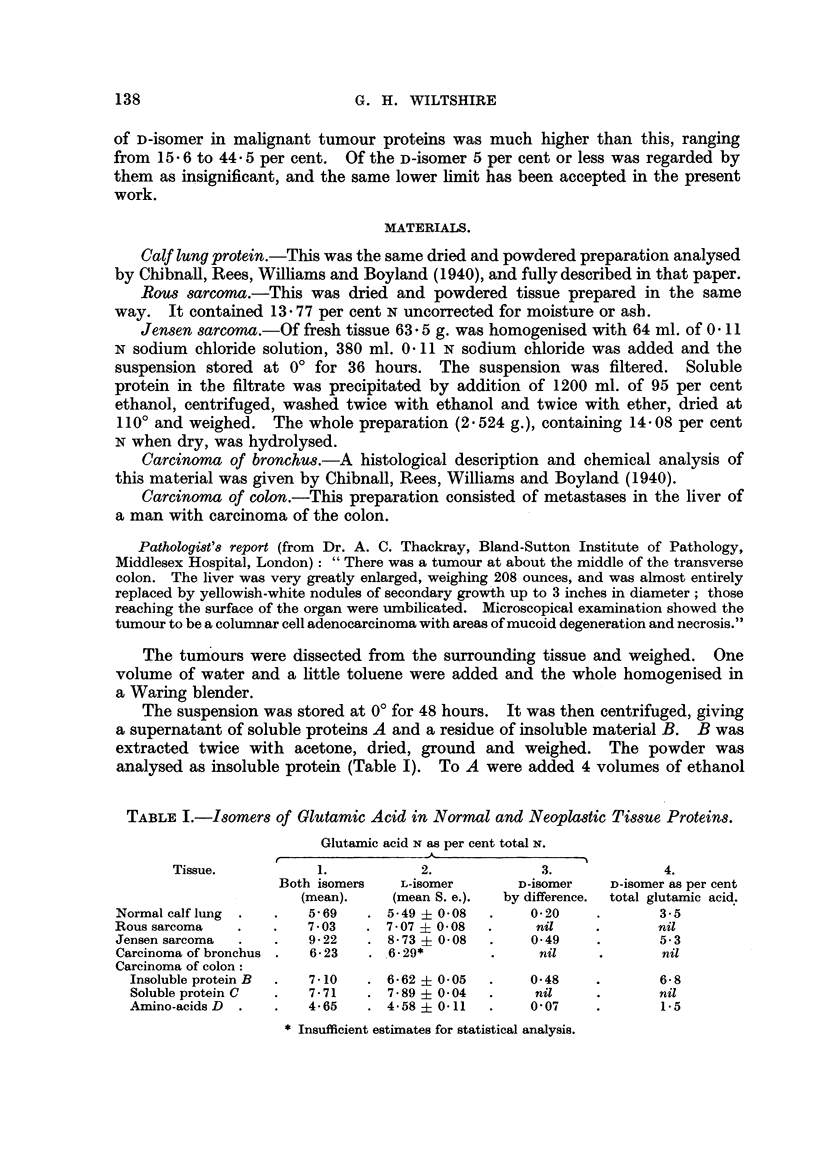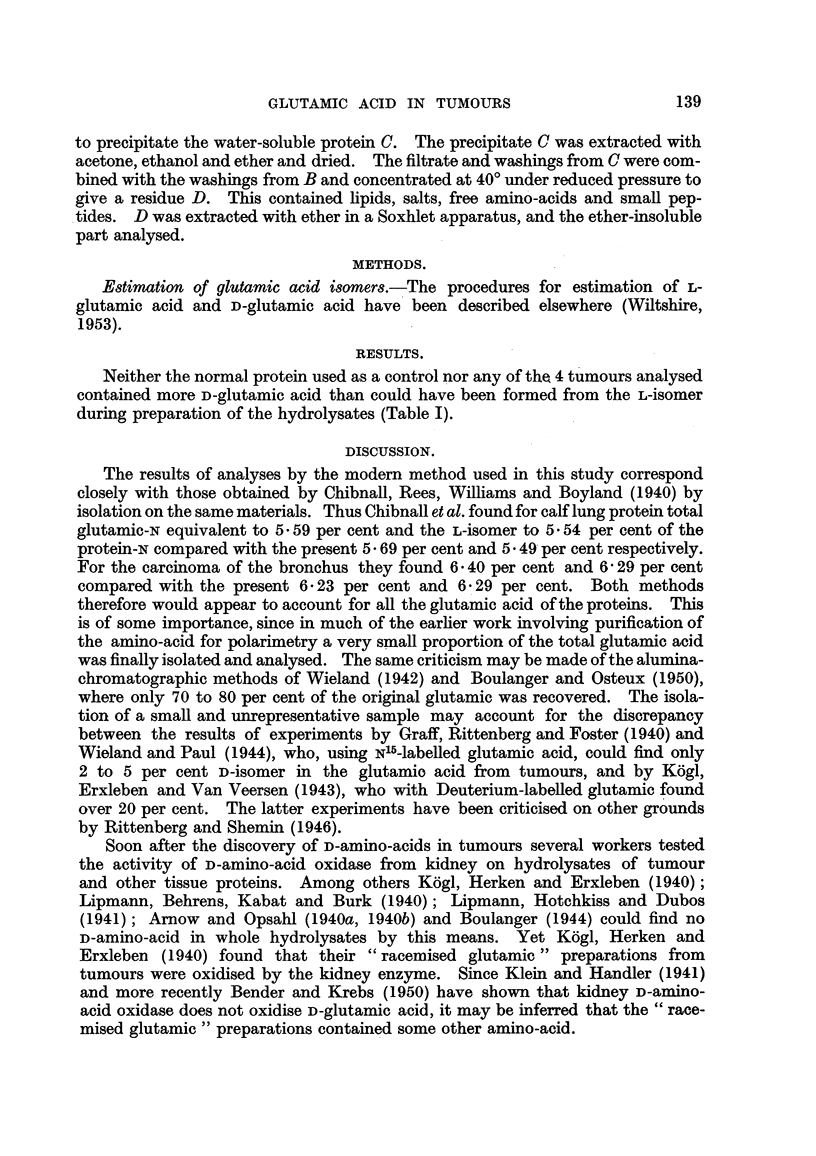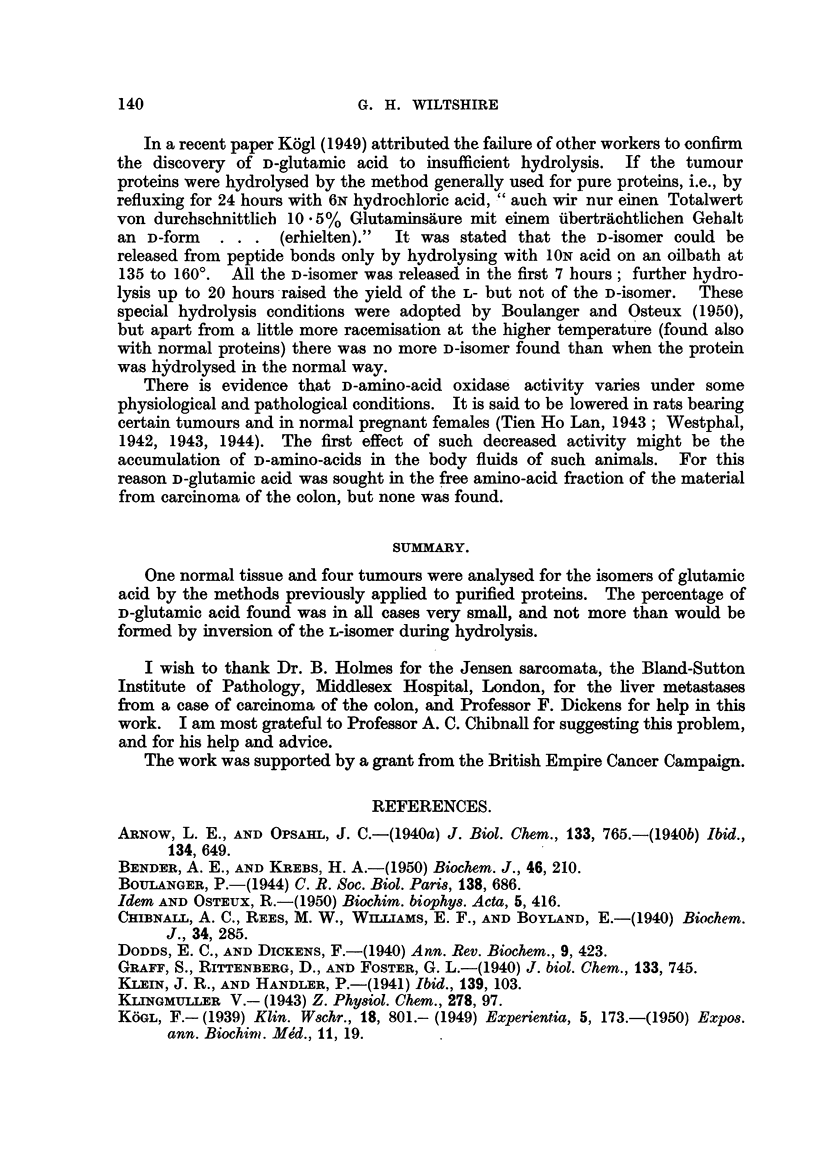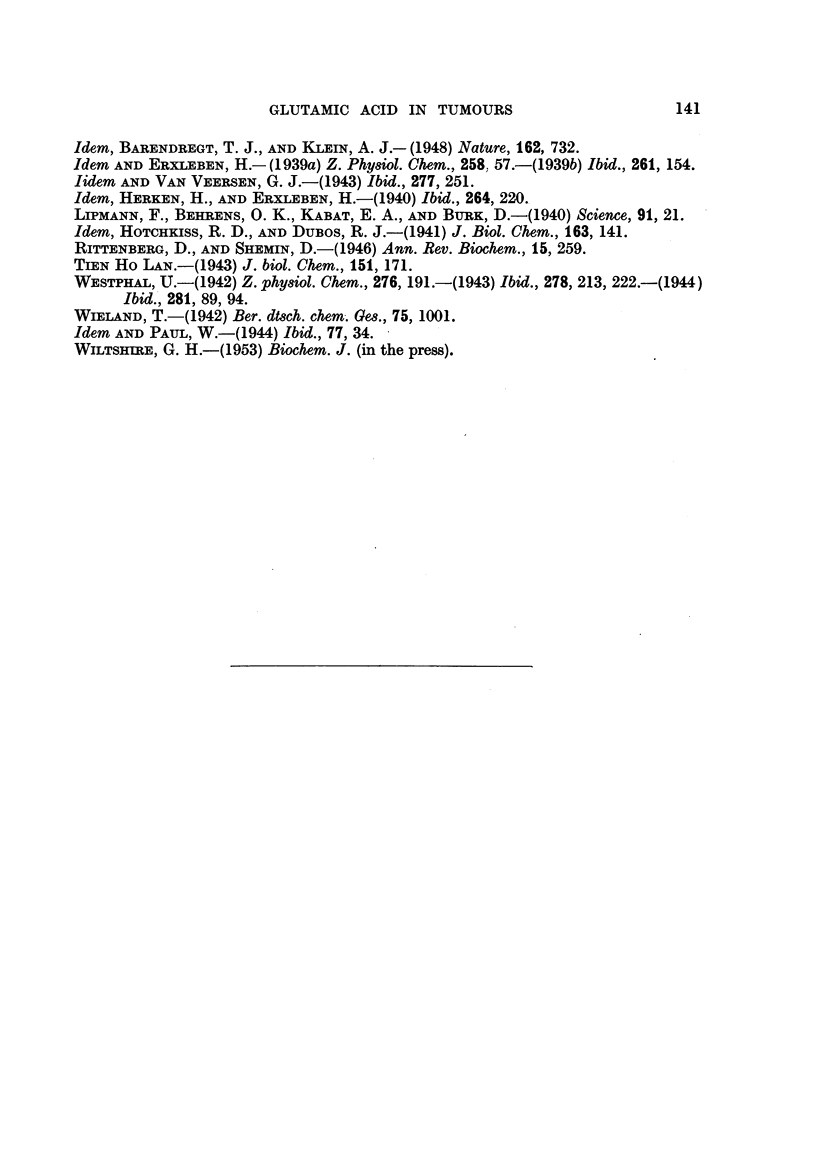# The Optical From of Glutamic Acid in Tumours

**DOI:** 10.1038/bjc.1953.12

**Published:** 1953-03

**Authors:** G. H. Wiltshire


### THE OPTICAL FORM OF GLUTAMIC ACID IN TUMOURS.

G. H. WILTSHIRE.

*From the Biochemical Laboratory, University of Cambridge.*

Received for publication January 22, 1953.

IN 1939 Kögl and Erxleben (1939*a*) published the first of a series of papers on the optical configuration of amino-acids in proteins from tumours. It was found that the optical rotations of some amino-acids, which had been isolated by the classical methods and purified by recrystallisation, did not correspond with the values in the literature. It was concluded that the preparations contained the "unnatural" D-isomer in addition to the "natural" or L-isomer. The calculated proportion of D-isomer was largest in serine and glutamic acid. The authors considered that serine had probably been partly racemised during hydrolysis of the proteins or during subsequent purification. Glutamic acid on the other hand was considered resistant to racemisation in acid solution. Glutamic acid from normal tissues and benign tumours had the specific rotation $[\alpha]_{D}^{20^\circ}$ + 31·6°. Attention was directed to this apparent difference in the optical form of glutamic acid from malignant and non-malignant tissues, which promised to be a lead to the long-sought chemical difference between normal and neoplastic tissue, and one that could be used for diagnostic purposes. A theory of malignity was proposed (Kögl, 1939) depending on the inability of most peptidases to hydrolyse peptides of D-amino-acids.

The experiments of Kögl and collaborators were repeated in many other laboratories with variable results, which on the whole showed smaller amounts of D-isomer than had been found by Kögl. An especially thorough investigation was made by Klingmuller (1943). The considerable controversy which developed between different schools was suspended by the recent war, but Dodds and Dickens (1940) had already concluded from a review of the results then available that the appearance of the D-isomer of glutamic acid was not characteristic of tumour tissues. Since the war further work by Kögl, Barendregt and Klein (1948) and Kögl (1949, 1950) has renewed discussion of this problem. Indirect evidence was produced in support of the original hypothesis. It was decided to limit the present study to the problem of whether or not the D-isomer is found in protein hydrolysates, and if so whether it exists as such in the proteins or is an artifact. A similar study using different methods (Boulanger and Osteux, 1950) has appeared since the experimental work was completed.

Preliminary experiments (Wiltshire, 1953) showed that glutamic acid is racemised to the extent of about 5 per cent in the preparation of protein hydrolysates. Normal plant and animal protein hydrolysates contained up to 6 per cent of the D-isomer. Kögl and Erxleben (1939*b*) claimed that the proportion of D-isomer in malignant tumour proteins was much higher than this, ranging from 15·6 to 44·5 per cent. Of the D-isomer 5 per cent or less was regarded by them as insignificant, and the same lower limit has been accepted in the present work.

### MATERIALS.

*Calf lung protein.*—This was the same dried and powdered preparation analysed by Chibnall, Rees, Williams and Boyland (1940), and fully described in that paper.

*Rous sarcoma.*—This was dried and powdered tissue prepared in the same way. It contained 13·77 per cent N uncorrected for moisture or ash.

*Jensen sarcoma.*—Of fresh tissue 63·5 g. was homogenised with 64 ml. of 0·11 N sodium chloride solution, 380 ml. 0·11 N sodium chloride was added and the suspension stored at 0° for 36 hours. The suspension was filtered. Soluble protein in the filtrate was precipitated by addition of 1200 ml. of 95 per cent ethanol, centrifuged, washed twice with ethanol and twice with ether, dried at 110° and weighed. The whole preparation (2·524 g.), containing 14·08 per cent N when dry, was hydrolysed.

*Carcinoma of bronchus.*—A histological description and chemical analysis of this material was given by Chibnall, Rees, Williams and Boyland (1940).

*Carcinoma of colon.*—This preparation consisted of metastases in the liver of a man with carcinoma of the colon.

*Pathologist's report* (from Dr. A. C. Thackray, Bland-Sutton Institute of Pathology, Middlesex Hospital, London) : " There was a tumour at about the middle of the transverse colon. The liver was very greatly enlarged, weighing 208 ounces, and was almost entirely replaced by yellowish-white nodules of secondary growth up to 3 inches in diameter ; those reaching the surface of the organ were umbilicated. Microscopical examination showed the tumour to be a columnar cell adenocarcinoma with areas of mucoid degeneration and necrosis."

The tumours were dissected from the surrounding tissue and weighed. One volume of water and a little toluene were added and the whole homogenised in a Waring blender.

The suspension was stored at 0° for 48 hours. It was then centrifuged, giving a supernatant of soluble proteins *A* and a residue of insoluble material *B*. *B* was extracted twice with acetone, dried, ground and weighed. The powder was analysed as insoluble protein (Table I). To *A* were added 4 volumes of ethanol

TABLE I.—*Isomers of Glutamic Acid in Normal and Neoplastic Tissue Proteins.*

| Tissue. | Glutamic acid N as per cent total N. 1. Both isomers (mean). | 2. L-isomer (mean S. e.). | 3. D-isomer by difference. | 4. D-isomer as per cent total glutamic acid. |
|---|---|---|---|---|
| Normal calf lung | 5·69 | 5·49 ± 0·08 | 0·20 | 3·5 |
| Rous sarcoma | 7·03 | 7·07 ± 0·08 | *nil* | *nil* |
| Jensen sarcoma | 9·22 | 8·73 ± 0·08 | 0·49 | 5·3 |
| Carcinoma of bronchus | 6·23 | 6·29* | *nil* | *nil* |
| Carcinoma of colon : | | | | |
| Insoluble protein *B* | 7·10 | 6·62 ± 0·05 | 0·48 | 6·8 |
| Soluble protein *C* | 7·71 | 7·89 ± 0·04 | *nil* | *nil* |
| Amino-acids *D* | 4·65 | 4·58 ± 0·11 | 0·07 | 1·5 |

* Insufficient estimates for statistical analysis.

to precipitate the water-soluble protein *C*. The precipitate *C* was extracted with acetone, ethanol and ether and dried. The filtrate and washings from *C* were combined with the washings from *B* and concentrated at 40° under reduced pressure to give a residue *D*. This contained lipids, salts, free amino-acids and small peptides. *D* was extracted with ether in a Soxhlet apparatus, and the ether-insoluble part analysed.

### METHODS.

*Estimation of glutamic acid isomers.*—The procedures for estimation of L-glutamic acid and D-glutamic acid have been described elsewhere (Wiltshire, 1953).

### RESULTS.

Neither the normal protein used as a control nor any of the 4 tumours analysed contained more D-glutamic acid than could have been formed from the L-isomer during preparation of the hydrolysates (Table I).

### DISCUSSION.

The results of analyses by the modern method used in this study correspond closely with those obtained by Chibnall, Rees, Williams and Boyland (1940) by isolation on the same materials. Thus Chibnall *et al.* found for calf lung protein total glutamic-N equivalent to 5·59 per cent and the L-isomer to 5·54 per cent of the protein-N compared with the present 5·69 per cent and 5·49 per cent respectively. For the carcinoma of the bronchus they found 6·40 per cent and 6·29 per cent compared with the present 6·23 per cent and 6·29 per cent. Both methods therefore would appear to account for all the glutamic acid of the proteins. This is of some importance, since in much of the earlier work involving purification of the amino-acid for polarimetry a very small proportion of the total glutamic acid was finally isolated and analysed. The same criticism may be made of the alumina-chromatographic methods of Wieland (1942) and Boulanger and Osteux (1950), where only 70 to 80 per cent of the original glutamic was recovered. The isolation of a small and unrepresentative sample may account for the discrepancy between the results of experiments by Graff, Rittenberg and Foster (1940) and Wieland and Paul (1944), who, using $N^{15}$-labelled glutamic acid, could find only 2 to 5 per cent D-isomer in the glutamic acid from tumours, and by Kögl, Erxleben and Van Veersen (1943), who with Deuterium-labelled glutamic found over 20 per cent. The latter experiments have been criticised on other grounds by Rittenberg and Shemin (1946).

Soon after the discovery of D-amino-acids in tumours several workers tested the activity of D-amino-acid oxidase from kidney on hydrolysates of tumour and other tissue proteins. Among others Kögl, Herken and Erxleben (1940); Lipmann, Behrens, Kabat and Burk (1940); Lipmann, Hotchkiss and Dubos (1941); Arnow and Opsahl (1940*a*, 1940*b*) and Boulanger (1944) could find no D-amino-acid in whole hydrolysates by this means. Yet Kögl, Herken and Erxleben (1940) found that their "racemised glutamic" preparations from tumours were oxidised by the kidney enzyme. Since Klein and Handler (1941) and more recently Bender and Krebs (1950) have shown that kidney D-amino-acid oxidase does not oxidise D-glutamic acid, it may be inferred that the "racemised glutamic" preparations contained some other amino-acid.

In a recent paper Kögl (1949) attributed the failure of other workers to confirm the discovery of D-glutamic acid to insufficient hydrolysis. If the tumour proteins were hydrolysed by the method generally used for pure proteins, i.e., by refluxing for 24 hours with 6N hydrochloric acid, " auch wir nur einen Totalwert von durchschnittlich 10·5% Glutaminsäure mit einem überträchtlichen Gehalt an D-form . . . (erhielten)." It was stated that the D-isomer could be released from peptide bonds only by hydrolysing with 10N acid on an oilbath at 135 to 160°. All the D-isomer was released in the first 7 hours; further hydrolysis up to 20 hours raised the yield of the L- but not of the D-isomer. These special hydrolysis conditions were adopted by Boulanger and Osteux (1950), but apart from a little more racemisation at the higher temperature (found also with normal proteins) there was no more D-isomer found than when the protein was hydrolysed in the normal way.

There is evidence that D-amino-acid oxidase activity varies under some physiological and pathological conditions. It is said to be lowered in rats bearing certain tumours and in normal pregnant females (Tien Ho Lan, 1943; Westphal, 1942, 1943, 1944). The first effect of such decreased activity might be the accumulation of D-amino-acids in the body fluids of such animals. For this reason D-glutamic acid was sought in the free amino-acid fraction of the material from carcinoma of the colon, but none was found.

### SUMMARY.

One normal tissue and four tumours were analysed for the isomers of glutamic acid by the methods previously applied to purified proteins. The percentage of D-glutamic acid found was in all cases very small, and not more than would be formed by inversion of the L-isomer during hydrolysis.

I wish to thank Dr. B. Holmes for the Jensen sarcomata, the Bland-Sutton Institute of Pathology, Middlesex Hospital, London, for the liver metastases from a case of carcinoma of the colon, and Professor F. Dickens for help in this work. I am most grateful to Professor A. C. Chibnall for suggesting this problem, and for his help and advice.

The work was supported by a grant from the British Empire Cancer Campaign.

### REFERENCES.

Arnow, L. E., and Opsahl, J. C.—(1940*a*) *J. Biol. Chem.*, **133**, 765.—(1940*b*) *Ibid.*, **134**, 649.

Bender, A. E., and Krebs, H. A.—(1950) *Biochem. J.*, **46**, 210.

Boulanger, P.—(1944) *C. R. Soc. Biol. Paris*, **138**, 686.

*Idem* and Osteux, R.—(1950) *Biochim. biophys. Acta*, **5**, 416.

Chibnall, A. C., Rees, M. W., Williams, E. F., and Boyland, E.—(1940) *Biochem. J.*, **34**, 285.

Dodds, E. C., and Dickens, F.—(1940) *Ann. Rev. Biochem.*, **9**, 423.

Graff, S., Rittenberg, D., and Foster, G. L.—(1940) *J. biol. Chem.*, **133**, 745.

Klein, J. R., and Handler, P.—(1941) *Ibid.*, **139**, 103.

Klingmuller V.— (1943) *Z. Physiol. Chem.*, **278**, 97.

Kögl, F.— (1939) *Klin. Wschr.*, **18**, 801.— (1949) *Experientia*, **5**, 173.—(1950) *Expos. ann. Biochim. Méd.*, **11**, 19.

*Idem*, BARENDREGT, T. J., AND KLEIN, A. J.—(1948) *Nature*, **162**, 732.
*Idem* AND ERXLEBEN, H.—(1939*a*) *Z. Physiol. Chem.*, **258**, 57.—(1939*b*) *Ibid.*, **261**, 154.
*Iidem* AND VAN VEERSEN, G. J.—(1943) *Ibid.*, **277**, 251.
*Idem*, HERKEN, H., AND ERXLEBEN, H.—(1940) *Ibid.*, **264**, 220.
LIPMANN, F., BEHRENS, O. K., KABAT, E. A., AND BURK, D.—(1940) *Science*, **91**, 21.
*Idem*, HOTCHKISS, R. D., AND DUBOS, R. J.—(1941) *J. Biol. Chem.*, **163**, 141.
RITTENBERG, D., AND SHEMIN, D.—(1946) *Ann. Rev. Biochem.*, **15**, 259.
TIEN HO LAN.—(1943) *J. biol. Chem.*, **151**, 171.
WESTPHAL, U.—(1942) *Z. physiol. Chem.*, **276**, 191.—(1943) *Ibid.*, **278**, 213, 222.—(1944) *Ibid.*, **281**, 89, 94.
WIELAND, T.—(1942) *Ber. dtsch. chem. Ges.*, **75**, 1001.
*Idem* AND PAUL, W.—(1944) *Ibid.*, **77**, 34.
WILTSHIRE, G. H.—(1953) *Biochem. J.* (in the press).